# Is the use of standardized patients more effective than role-playing in medical education? A meta-analysis

**DOI:** 10.3389/fmed.2025.1601116

**Published:** 2025-06-18

**Authors:** Jingyuan Xiao, Xinjian Fu

**Affiliations:** Guangzhou Xinhua University, Guangzhou, China

**Keywords:** role-playing, standardized patients, meta-analysis, medical education, clinical simulation

## Abstract

**Aim:**

This study aimed to compare the effectiveness of using Standardized patients (SPs) and Role-playing (RP) in medical education. It is crucial to understand the differences in the effects of SPs and RP. However, the existing measurement results are varied, and the findings lack robustness.

**Methods:**

We collected the results of various experiments and conducted a meta-analysis. In total, 10 articles and 27 effect sizes were included in the analysis, involving 721 students.

**Results:**

The meta-analysis results showed that compared to the RP method, using SPs significantly improved students’ self-confidence (effect size = 0.415). However, in other aspects, the two methods showed similar outcomes. We observed that the effectiveness of SPs teaching methods increased over time.

**Conclusion:**

SPs effectively enhance students’ self-confidence by simulating diverse roles, situations, and real-world work scenarios. This study provides a comprehensive comparative perspective on RP and SPs.

## 1 Introduction

The rapid development of economic globalization has driven the transformation of the medical field (1). As a result, the demands on medical workers have risen significantly, and their tasks have become increasingly complex (2). The traditional teaching method keeps medical students away from frontline work and fails to mobilize students’ enthusiasm (3). Therefore, it is essential to provide students with scenario-based and experiential teaching methods.

Standardized Patients (SPs) and Role-Playing (RP) are commonly used teaching methods in medicine and education (4). SPs are trained individuals who simulate various medical cases and clinical scenarios (5). Through interactions with SPs, students can practice physical examination, diagnosis, communication, patient management, and medical decision-making skills. RP allows learners to assume roles and engage with different situations to enhance their skills and cognition (6). As a situational simulation teaching method, RP has been widely applied across various disciplines and fields (7). In medical education, both RP and SP methods represent a shift from a teacher-centered to student-centered approach. These methods simulate clinical settings, helping students apply theory and build skills like observation and communication.

At present, there have been many studies related to SPs and RP, such as the situational strategy and the reshaping strategy of learning interest (8). Different research environments, student groups, and implementation details may lead to differences in the effectiveness of the strategies in different contexts (9). Although existing studies have examined SPs and RP separately, there is still no clear consensus on which approach is more effective. This study aims to evaluate the relative effectiveness of SPs and RP in medical education. Specifically, it examines whether SPs are more effective than RP overall, and whether significant differences exist between the two methods across various dimensions of learning outcomes.

## 2 Literature review

Many scholars have explored the impact of medical education using SPs. For instance, Ross et al. (10) found that combining SPs with lectures effectively educated nursing students about elder abuse. Similarly, Kim and Kim (11) reported that an 8-h psychiatric nursing simulation significantly improved students’ self-directed learning and self-efficacy. Webster (12) also confirmed the effectiveness of SPs in enhancing therapeutic communication skills. Ok et al. (13) showed that SPs reduced anxiety and improved communication in mental health training, while Ha (14) highlighted improvements in students’ self-confidence, nursing abilities, and interest in learning.

While the benefits of SPs are well-documented, some scholars have focused on RP as an alternative method that can also improve student learning. RP provides simulated scenarios that aim to improve students’ empathy and emotional intelligence. However, some studies have questioned the effectiveness of RP. Bayne (15) argued that regulatory factors might hinder learning outcomes, while Delnavaz et al. (16) reported no significant improvements in students’ skills or theoretical knowledge. Lee and Kim (17) similarly found no notable gains in emotional intelligence.

Despite the independent use of either SPs or RP in many studies, both methods share similarities. Several studies have compared the effectiveness of these two methods. Mounsey et al. (18) observed no significant differences between RP and SPs in post-teaching videos. Yeung (19) found that RP and SPs differed in their effects on self-efficacy but not on performance in adverse event disclosure training. Taylor et al. (20) and Schlegel et al. (21) both reported no significant differences between RP and SP in terms of communication skills and self-efficacy, respectively, suggesting comparable effectiveness in these domains.

Scholars have also conducted a literature review of SPs and RP. For example, Ma et al. (22) found that SP simulations significantly enhanced nursing students’ communication, self-efficacy, problem-solving, and learning satisfaction. Gelis et al. (23) showed that RP effectively improved communication skills and was more cost-effective than SPs. Dalwood et al. (24) found that peer simulation, where healthcare students role-play as patients, was more effective in enhancing empathy. Chua et al. (25) further supported this by demonstrating that simulation-based interventions effectively enhanced medical students’ empathy.

Although some systematic reviews have examined RP or SPs individually, few have compared the effectiveness of the two methods. Meta-analysis enables the systematic synthesis of empirical studies to obtain comprehensive effect estimates. Accordingly, this study employs a meta-analytic approach to examine the differences in the effectiveness of RP and SPs in medical education. The results can inform educators’ decisions in selecting appropriate instructional methods to enhance teaching outcomes.

## 3 Research design

### 3.1 Research methods and tools

A meta-analysis is a statistical method used to synthesize and integrate results from multiple independent studies (26). By combining data from various sources, it enables researchers to draw more comprehensive and reliable conclusions (27), while reducing the instability caused by small sample sizes or random errors in individual studies. This approach also incorporates a broader range of samples, leading to more robust and generalizable findings. Given these advantages, this study adopts meta-analysis as the primary research method. Comprehensive Meta-Analysis (CMA) software, a specialized tool for conducting meta-analyses, provides an all-in-one solution for data analysis (28). In addition to its analytical functions, CMA offers tools for testing heterogeneity and assessing bias, making it well-suited for this study’s data processing and analysis.

### 3.2 Search strategy

The procedure for selecting studies followed the guidelines of the Preferred Reporting Items for Systematic Reviews and Meta-Analysis (29). The literature analyzed in this study was primarily sourced from databases such as EBSCO, ProQuest, ScienceDirect, SpringerLink, Web of Science, and PubMed. The literature search utilized two main groups of keywords: (1) RP-related keywords, including “role-play,” “role-playing,” “role playing,” and “RP”; (2) SP-related keywords, including “standardized patient,” “SP,” and “SPs”; and (3) keywords related to outcomes, such as “performance,” “effectiveness,” “effect,” “achievement,” and “outcome.” Boolean operators, “AND” and “OR,” were used to combine keywords both within and between groups, respectively.

### 3.3 Eligibility criteria

**FIGURE 1:**
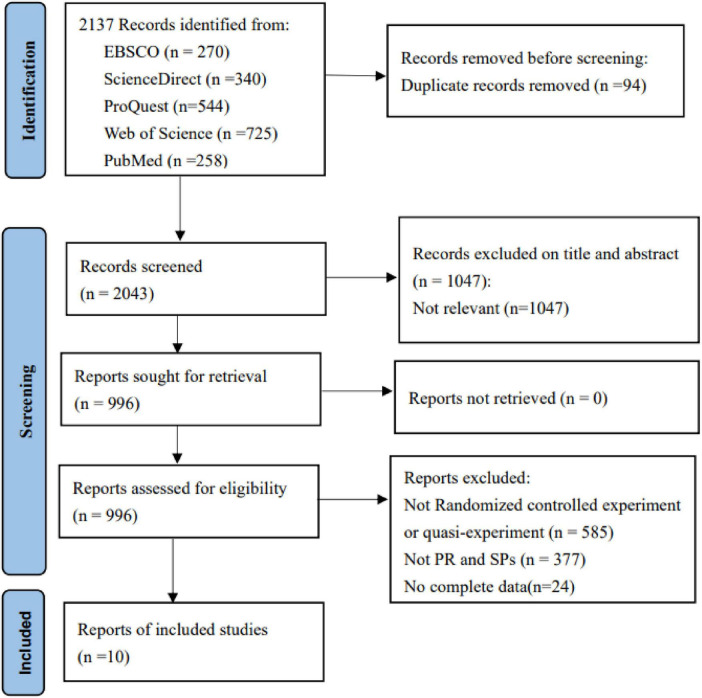
Flowchart of literature inclusion.

The literature was screened according to the following criteria: (1) The study employed an experimental or quasi-experimental design; (2) Both an experimental group and a control group were included, with the intervention involving the use of RP and SPs in teaching; (3) Sufficient statistical data were provided, including sample sizes, means, variances, and other relevant metrics for both groups; (4) The participants were medical personnel. Based on these criteria, 2, 137 papers were retrieved and screened, with the process shown in Figure 1. After thoroughly reviewing the titles, abstracts and full texts, the two researchers collaborated to identify 10 studies that would be included in the meta-analysis.

### 3.4 Quality appraisal

Among the included studies, five were quasi-experiments and five were randomized controlled trials (RCTs). Each study was independently reviewed by two investigators. The quasi-experimental studies were evaluated using the JBI Checklist for Quasi-Experimental Studies, which includes nine items with four response options: “Yes,” “No,” “Unclear,” and “Not Applicable.” For the RCTs, the Risk of Bias 2 (RoB 2) tool was employed. This tool assesses the risk of bias across five domains: selection bias, performance bias, detection bias, attrition bias, and reporting bias. Each domain was rated as having a “low risk,” “high risk,” or “unclear risk” of bias.

### 3.5 Data encoding and effect size

After the literature retrieval, two independent reviewers coded the characteristics of the collected original literature (30). Two independent reviewers coded the included studies based on a predefined coding framework. Any discrepancies between the reviewers were resolved through joint consultation. Inter-rater reliability was assessed using Cohen’s Kappa, with a value of 0.81 indicating substantial agreement. The coding categories of this study included author, publication year, experiment type, sample size, publication type, country, and learning effectiveness type. The experiments were classified into two types: RCTs (Randomized Controlled Trials) and quasi-experiments. Sample sizes were categorized into three groups: small (≤ 50 participants), medium (51 The experiments were classifi(> 100 participants). The publication types were categorized into two groups: journal articles and theses. The studies were conducted in Germany, Indonesia, Korea, China, and the United States. The learning outcomes examined in this study include communication, emotional response, overall performance, knowledge acquisition, self-efficacy, professional competence, and self-confidence. Detailed coding information is presented in Table 1.

**TABLE 1:** Summary of included studies.

Author, year	Study design	Sample size	Publication type	Sample region	Outcome type
Bosse et al. (34)	RCTs	Medium	Article	Germany	Self-efficacy
Hans et al. (35)	RCTs	Medium	Article	Germany	Self-efficacy, general performance
Cahyono et al. (36)	Quasi experiment	Small	Article	Indonesia	Self-confidence, communication
Kim et al. (37)	Quasi experiment	Medium	Article	Korea	Knowledge
Park et al. (38)	RCTs	Large	Article	Korea	Knowledge, self-confidence, general performance
Yeung (19)	Quasi experiment	Small	Article	China	Communication, professional competence, emotions, self-efficacy
Yan (39)	Quasi experiment	Large	Thesis	China	Communication, professional competence, emotions, self-efficacy
Li et al. (40)	Quasi experiment	Small	Article	China	Self-confidence
Bradford (41)	RCTs	Medium	Thesis	America	Self-confidence, general performance
Lupiani (42)	RCTs	Medium	Thesis	America	Self-efficacy

Effect sizes are statistical indicators that quantify the magnitude of differences or associations between variables or treatments of interest (31). Different disciplines often employ different effect size metrics (32). In educational research, commonly used measures include the standardized mean difference (SMD), as well as Cohen’s *d* and Hedges’ *g*. While Cohen’s *d* may overestimate effect sizes in small samples (33), Hedges’ g includes a correction for small sample bias, making it more appropriate for studies with limited sample sizes (19). Given the relatively small sample sizes in the included studies, this meta-analysis adopted Hedges’ g as the effect size metric.

## 4 Results

### 4.1 Risk of bias in studies

**FIGURE 2:**
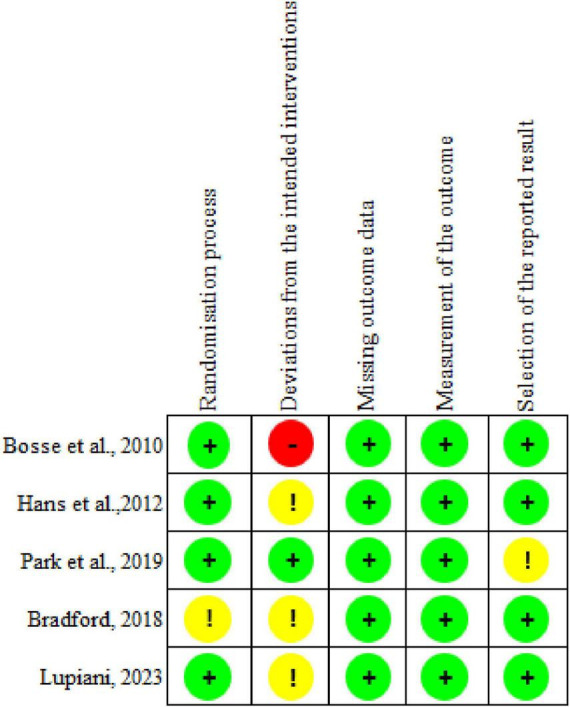
Assessment of the risk of bias in the included articles.

Using the JBI checklist for assessment, most studies were found to have a low risk of bias, as shown in Table 2. The RoB2 assessment identified four studies with a low risk in the randomization process. However, many studies lacked allocation concealment and random assignment, largely due to the nature of topic selection. Regarding deviations from the intended interventions, one study had a low risk, three raised some concerns, and one was classified as high risk. This was primarily because both participants and intervention providers were generally aware of the intervention. For missing outcome data, five studies demonstrated a low risk, with no major issues identified. All studies showed a low risk in outcome measurement, as they employed rigorous analytical methods. Finally, four studies were rated as having a low risk in the selection of reported results, as illustrated in Figure 2.

**TABLE 2:** Quality assessment of the included studies of quasi-experimental study.

Studies	Questions								
	1	2	3	4	5	6	7	8	9
Cahyono et al. (36)	Y	Y	Y	Y	N	N	Y	Y	Y
Kim et al. (37)	Y	Y	Y	Y	Y	N	Y	Y	Y
Yeung (19)	Y	Y	Y	Y	Y	N	Y	Y	Y
Li et al. (40)	Y	Y	Y	Y	Y	N	Y	Y	Y
Yan (39)	Y	Y	Y	Y	Y	N	Y	Y	Y

### 4.2 Test for bias

Publication bias refers to the tendency for studies with statistically significant findings to be more likely published (43). In contrast, non-significant results may remain unpublished due to editorial decisions or authors’ self-selection, leading to incomplete or skewed representations of the research landscape. To ensure the scientific validity of meta-analytic results, it is therefore essential to assess and account for publication bias in the primary studies included in the analysis.

**FIGURE 3:**
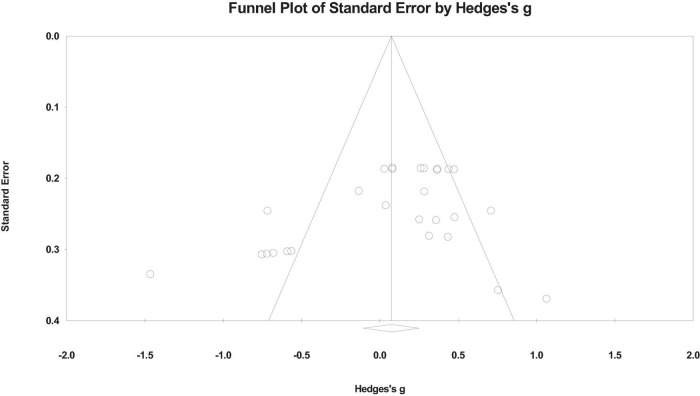
The funnel plot shows publication bias among outcome type the included studies. Each point represents a study, distributed around the center line.

This study employed funnel plots as well as Begg’s tests to assess publication bias (44). Funnel plots visually help detect publication bias. As shown in Figure 3, the funnel plot for the effect size of understanding level is symmetrically distributed around the axis, with *g* = 0.09. Additionally, Begg and Mazumdar’s rank correlation test (Kendall’s τ = –0.179, *p* = 0.18) and Egger’s regression test for the intercept [intercept = –3.189, 95% CI (–6.631, 0.251), *t* = 1.90, df = 25, *p* = 0.067] yielded *p*-values greater than 0.05. These results suggest that there is no significant publication bias in the sample, supporting its use for further analysis.

### 4.3 Heterogeneity test

Heterogeneity tests assess the extent to which effect sizes vary across independent studies (45). These tests determine whether significant differences exist between studies’ effect sizes and whether it is appropriate to pool their results. The *Q* test and *I*^2^ statistic are commonly used to evaluate heterogeneity (46). If the *Q* value exceeds *K*−1 (where *K* is the number of effect sizes), and the *p*-value is ≤ 0.05, significant heterogeneity is indicated, and a random-effects model is recommended. An *I*^2^ value greater than 50% also suggests substantial heterogeneity (47). In the present study, the Q value was 100.963 (*p* < 0.05), and the *I*^2^ value was 74.248%. Given that the number of effect sizes was 27, the *Q* value exceeds the threshold of 26, and the *I*^2^ result confirms high heterogeneity. Therefore, a random-effects model was employed for analysis.

### 4.4 Overall effect test results

Based on the effect size interpretation framework proposed by Cohen (48), values above 0.2 are regarded as small, those exceeding 0.5 as medium, and values greater than 0.8 as large. Table 3 shows that the pooled effect size is 0.073, which does not reach statistical significance (*P* < 0.001), indicating no overall significant difference between using SPs and RP.

**TABLE 3:** Overall effect size.

			Test of null		Heterogeneity		
*K*	ES	95% Cl	*Z*	*p*	*Q*	*p*	*I* ^2^
27	0.073	[–0.104, 0.250]	0.810	0.418	100.963	0.000	74.248

### 4.5 Subgroup analysis of learning outcomes

Subgroup analyses related to learning effects are shown in Table 4. The effect sizes (ES) for the influence of the virtual patient teaching method on learners’ communication, emotions, general performance, knowledge, professional competence, self-confidence, and self-efficacy were as follows: 0.200 (*p* > 0.05), –0.115 (*p* > 0.05), –0.089 (*p* > 0.05), 0.221 (*p* > 0.05), –0.068 (*p* > 0.05), 0.415 (*p* < 0.001), and –0.087 (*p* > 0.05). In this analysis, communication refers to learners’ ability to engage effectively with patients, including active listening and clear verbal expression (49). Knowledge denotes the learners’ understanding and retention of medical information (50). Confidence captures learners’ self-perceived assurance in carrying out clinical tasks (51). Among these, only the effect on self-confidence reached statistical significance, albeit with a small effect size. The heterogeneity for self-confidence was 0, indicating no significant variability. The heterogeneity test for the subgroup comparisons of different types of learning effects showed no impact on variance (*Q*_*B*_ = 8.111, *p* > 0.05).

**TABLE 4:** Subgroup analyses related to learning effects.

				Heterogeneity		
**Outcome type**	***K***	**ES**	**95% CI**	***Q***	***I* ^2^**	
Communication	3	0.200	[–0.52, 0.92]	10.71	81.32**	
Emotions	2	–0.115	[–1.31, 1.08]	11.62	91.39***	
General performance	3	–0.089	[–0.67, 0.50]	10.31	80.61**	
Knowledge	2	0.221	[–0.21, 0.65]	2.00	50.14	Q_*B*_ = 8.111
Professional competence	2	–0.068	[–0.97, 0.84]	6.85	85.42**	
Self-confidence	5	0.415***	[0.18, 0.64]	3.69	0.00	
Self-efficacy	10	–0.087	[–0.40, 0.23]	44.38	79.72***	

**p < 0.01;

***p < 0.001. K represents the number of effect values included; ES stands for effect size; 95% CI represents the confidence interval of the level; Q represents intra-group heterogeneity test statistic; Q_*B*_ is the test statistic of inter-group heterogeneity. *I*^2^ indicates the degree of inconsistency between the results of different studies.

### 4.6 Meta-analysis related to publication type

Subgroup analyses related to publication type are presented in Table 5. The results of the group effect test showed that *Q*_*B*_ = 4.531, *p* < 0.05, indicating a significant difference in the impact across different publication types. Within the publication types, the ES for articles and theses were –0.065 (*p* > 0.05) and 0.274 (*p* < 0.001), respectively. Although the effect size for articles is negative, it is not statistically significant. However, the effect size for theses reaches statistical significance, with a small positive effect. The degree of heterogeneity for this comparison was 0, indicating no variability.

**TABLE 5:** Subgroup analysis with respect to publication type.

				Heterogeneity		
Publication type	*K*	ES	95% CI	*Q*	*I* ^2^	
Article	17	–0.065	[–0.35, 0.22]	83.65	80.872***	Q_*B*_ = 4.531*
Thesis	10	0.274***	[0.14, 0.39]	6.885	0.000	

*p < 0.05,

***p < 0.001.

### 4.7 Subgroup analysis related to sample size

Subgroup analysis concerning sample size is shown in Table 6. The results of the group effect test indicated that *Q*_*B*_ = 4.165, *p* > 0.05. The effect sizes for large, medium, and small sample sizes were 0.264 (*p* < 0.001), 0.155 (*p* > 0.05), and –0.228 (*p* > 0.05), respectively. Only the large sample size reached statistical significance and had a positive effect. Regarding heterogeneity, the degree of heterogeneity for the large sample size was 0, indicating no significant variability.

**TABLE 6:** Subgroup analysis with respect to sample size.

				Heterogeneity		
Sample size	*K*	ES	95% CI	*Q*	*I* ^2^	
Large	9	0.264***	[0.14, 0.38]	6.25	0.00	
Medium	8	0.155	[–0.14, 0.45]	22.38	68.72**	Q_*B*_ = 4.165
Small	10	–0.228	[–0.69, 0.24]	53.02	83.02***	

**p < 0.01,

***p < 0.001.

### 4.8 Analysis of subgroups related to countries

The subgroup analyses related to sample regions are shown in Table 7. The results of the group effect test indicated that *Q*_*B*_ = 12.101, *p* < 0.05, suggesting that the influence of different sample regions is significantly different. For the different sample regions, the ES for America, China, Germany, Indonesia, and Korea were 0.171 (*p* > 0.05), –0.102 (*p* > 0.05), 0.011 (*p* > 0.05), 0.906 (*p* < 0.001), and 0.209 (*p* < 0.05), respectively. Only the effect sizes for Indonesia and Korea reached statistical significance, with Indonesia showing a significant influence and Korea showing a small effect.

**TABLE 7:** Subgroup analysis related to the sample area.

				Heterogeneity		
Sample region	*K*	ES	95% CI	*Q*	*I* ^2^	
America	4	0.171	[–0.06, 0.40]	2.85	0.00	
China	14	–0.102	[–0.38, 0.17]	65.82	80.25	Q_*B*_ = 12.101*
Germany	3	0.011	[–0.78, 0.81]	16.87	88.14	
Indonesia	2	0.906***	[0.40, 1.40]	0.36	0.00	
Korea	4	0.209*	[0.01, 0.41]	3.19	6.10	

*p < 0.05,

***p < 0.001.

### 4.9 Cumulative analysis

**FIGURE 4:**
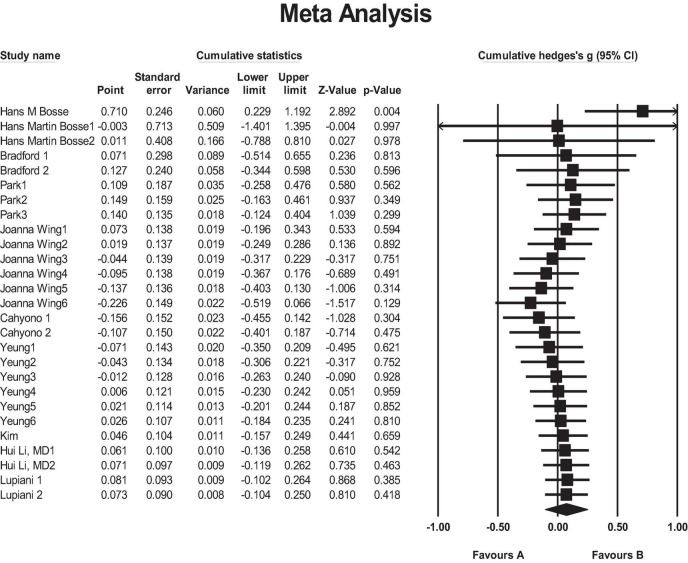
Forest plot of outcomes type for SPs compared with RP. Favors A, SPs; Favors B, RP.

Cumulative meta-analysis refers to a distinctive approach in which each original study is sequentially included in a meta-analysis, with the analysis updating as new data from individual studies are added. The most common method for accumulating studies is chronological, where the results demonstrate how the evidence has evolved over time. In this study, we assess the impact of the studies cumulatively based on the year of publication. The closer the value is to 1, the weaker the effect becomes. As shown in Figure 4, the effect size has exhibited an increasing trend over time.

## 5 Discussion

There was no significant difference between SPs and RP in terms of their overall impact on students. Both teaching methods had a similar effect. When considering the specific dimensions of learning outcomes, no significant differences were found between the two methods regarding students’ communication, emotions, general performance, knowledge, professional competence, and self-efficacy. However, SPs had a more positive effect on improving students’ self-confidence compared to the RP method, which aligns with the findings of Cahyono et al. (36). This may be attributed to the fact that SPs expose students to realistic clinical scenarios that simulate emotional exchanges between patients and healthcare providers. The realistic interactions involved in SPs help students develop greater confidence in their ability to navigate real-world clinical scenarios (52). Emotional agitation, anxiety, and tension are common in clinical environments (53). The use of SPs enables students to develop emotion regulation and coping strategies within simulated clinical environments. Such training helps learners remain composed during emergencies and respond more effectively to patients’ emotional needs. For medical students who have not yet experienced real clinical settings, interacting with actual patients can cause anxiety and uncertainty (54). SPs training helps students gradually adapt to clinical roles, gain experience, and face real patients with more confidence during this transition (55). Through repeated interactions with SPs, students begin to perceive themselves as future healthcare professionals. This internalization of their professional identity plays a critical role in strengthening their self-confidence in clinical practice (56). These experiences have been shown to significantly enhance students’ self-confidence in real-world clinical practice. However, it is noteworthy that this improvement was domain-specific. SPs did not yield significantly greater benefits than RP in other learning domains, possibly because these outcomes demand broader or more sustained forms of engagement beyond confidence-building alone.

Some studies were excluded from the analysis due to concerns about potential contributions to heterogeneity. To further explore this issue, publication types were categorized as either “articles” or “theses,” and a subgroup analysis was conducted. The results indicated that SPs had a significantly greater positive effect in theses compared to other methods. In contrast, studies published as articles showed no significant difference between SPs and RP, possibly due to the limited number of studies in this subgroup. Regarding teaching implementation, sample size did not appear to significantly affect instructional outcomes overall. However, larger sample sizes were associated with more favorable results, particularly for SPs. This may be due to the inherent challenges of maintaining classroom discipline and engagement during RP activities in large groups, where instructors may struggle to effectively manage the learning environment, thereby reducing RP’s instructional effectiveness compared to SPs.

Significant differences were observed across different sample areas. Our analysis included studies conducted in five countries: the United States, China, Germany, Indonesia, and Korea. Among these, Indonesia showed the most significant effect, with an effect size of 0.906, which was statistically significant at the 0.1% level. However, this finding should be interpreted cautiously due to the small number of Indonesian studies. Overgeneralizing from such limited data may lead to inaccurate conclusions. Cumulative analysis showed that the effect size was initially small and highly variable but gradually increased over time, indicating an overall upward trend. This pattern may reflect improvements in research design and methodology over the years. As studies adopted more rigorous controls, the reported effect sizes became more stable and pronounced. In earlier periods, studies with small or non-significant effects were less likely to be published—a phenomenon known as the “file drawer effect.” However, growing emphasis on study registration and research transparency in recent years has helped reduce publication bias and expanded the range of studies available for cumulative analysis.

In order to optimize the use of standardized patients, clear learning objectives should be defined prior to implementation (57). These objectives should be aligned with course goals to ensure appropriate scenario selection and meaningful performance evaluation. Our findings suggest that SPs is particularly effective in enhancing students’ self-confidence. Therefore, when confidence-building is a central instructional goal, SPs should be prioritized when feasible. During course sessions, educators should guide the activity through timely, constructive feedback (58), helping students reflect and improve without disrupting their immersion.

The duration of simulation-based teaching should be carefully planned and adjusted based on the complexity of each scenario (59). More complex situations may require longer sessions and extended discussion time to ensure students can fully comprehend and apply relevant knowledge and skills (60). Each phase should be appropriately timed to avoid the negative effects of excessively long or short sessions on learning outcomes. Flexibility in time management is essential (61). For instance, if students are actively engaged, extending the session may enhance reflection and learning. Conversely, if students appear fatigued or time is limited, the session can be shortened. Clearly defined learning objectives help instructors select suitable scenarios and roles and assess whether student performance meets expectations. Teachers should also be mindful of when to intervene, avoiding interruptions during moments of deep engagement, as this may disrupt the learning process.

Given the variability in students’ learning preferences and responsiveness, educators should consider different instructional approaches (62). Some students may benefit more from RP, while others respond better to SPs. Thus, teaching methods should be selected and adapted based on students’ needs and the complexity of the course content.

## 6 Limitations

This study has the following advantages: first, the experimental data were retrieved from multiple major databases from the establishment of the study up to 2024. Secondly, the included studies are of high quality. We also conducted a subgroup analysis, which partially explains the sources of heterogeneity in this study.

We have to admit that the study has some limitations. The limitation is related to the study sample, which is a common issue in almost all meta-analyses. For instance, in our subgroup analysis based on sample region, only two studies from Indonesia were included. The small number of studies in this subgroup may lead to less precise estimates. In the future, some unpublished literature needs to be included, and as the number of studies increases, this problem can be solved more effectively. The second limitation is that this study considered only a limited set of variables that may affect the effectiveness of the intervention. Important factors such as gender differences and prior frontline work experience were not included, although they may significantly influence student outcomes. This omission was primarily due to the fact that many of the included studies did not report these variables in sufficient detail. Future research should aim to incorporate such factors to provide a more comprehensive understanding of their potential impact on intervention effectiveness. Finally, language bias may be present, as most studies were conducted in English. Future research could include studies in other languages to enhance the robustness of the overall findings.

## 7 Conclusion

This meta-analysis synthesized findings from studies comparing SP and RP methods in medical education. Our findings indicate that, in terms of overall impact, the effects of both methods on students are similar. However, SPs are more effective in improving self-confidence. Although this study offers a new comparative perspective, cultural and sample differences may limit the generalizability of the findings. While limitations remain, this study contributes meaningful evidence to the ongoing debate on the relative effectiveness of RP and SPs.

## Data Availability

The original contributions presented in the study are included in the article/supplementary material, further inquiries can be directed to the corresponding author.
